# OsHKT1;4-mediated Na^+^ transport in stems contributes to Na^+^ exclusion from leaf blades of rice at the reproductive growth stage upon salt stress

**DOI:** 10.1186/s12870-016-0709-4

**Published:** 2016-01-19

**Authors:** Kei Suzuki, Naoki Yamaji, Alex Costa, Eiji Okuma, Natsuko I. Kobayashi, Tatsuhiko Kashiwagi, Maki Katsuhara, Cun Wang, Keitaro Tanoi, Yoshiyuki Murata, Julian I. Schroeder, Jian Feng Ma, Tomoaki Horie

**Affiliations:** Division of Applied Biology, Faculty of Textile Science and Technology, Shinshu University, 3-15-1, Tokida, Ueda, Nagano 386-8567 Japan; Institute of Plant Science and Resources, Okayama University, Chuo 2-20-1, Kurashiki, 710-0046 Japan; Department of Biosciences, University of Milan, Via G. Celoria 26, 20133 Milan, Italy; Graduate School of Environmental and Life Science, Okayama University, 1-1-1 Tsushima-naka, Okayama, 700-8530 Japan; Graduate School of Agricultural and Life Sciences, The University of Tokyo, 1-1–1 Yayoi, Bunkyo-ku, Tokyo 113-8657 Japan; Division of Biological Sciences, Cell and Developmental Biology Section, University of California, SanDiego, La Jolla, CA 92093-0116 USA; Institute of Biophysics, Consiglio Nazionale delle Ricerche, Via G. Celoria 26, 20133 Milan, Italy

**Keywords:** Salinity stress, Na^+^ transport, HKT, Rice

## Abstract

**Background:**

Na^+^ exclusion from leaf blades is one of the key mechanisms for glycophytes to cope with salinity stress. Certain class I transporters of the high-affinity K^+^ transporter (HKT) family have been demonstrated to mediate leaf blade-Na^+^ exclusion upon salinity stress via Na^+^-selective transport. Multiple HKT1 transporters are known to function in rice (*Oryza sativa*). However, the ion transport function of OsHKT1;4 and its contribution to the Na^+^ exclusion mechanism in rice remain to be elucidated.

**Results:**

Here, we report results of the functional characterization of the OsHKT1;4 transporter in rice. OsHKT1;4 mediated robust Na^+^ transport in *Saccharomyces cerevisiae* and *Xenopus laevis* oocytes. Electrophysiological experiments demonstrated that OsHKT1;4 shows strong Na^+^ selectivity among cations tested, including Li^+^, Na^+^, K^+^, Rb^+^, Cs^+^, and NH_4_^+^, in oocytes. A chimeric protein, EGFP-OsHKT1;4, was found to be functional in oocytes and targeted to the plasma membrane of rice protoplasts. The level of *OsHKT1;4* transcripts was prominent in leaf sheaths throughout the growth stages. Unexpectedly however, we demonstrate here accumulation of *OsHKT1;4* transcripts in the stem including internode II and peduncle in the reproductive growth stage. Moreover, phenotypic analysis of *OsHKT1;4* RNAi plants in the vegetative growth stage revealed no profound influence on the growth and ion accumulation in comparison with WT plants upon salinity stress. However, imposition of salinity stress on the RNAi plants in the reproductive growth stage caused significant Na^+^ overaccumulation in aerial organs, in particular, leaf blades and sheaths. In addition, ^22^Na^+^ tracer experiments using peduncles of RNAi and WT plants suggested xylem Na^+^ unloading by OsHKT1;4.

**Conclusions:**

Taken together, our results indicate a newly recognized function of OsHKT1;4 in Na^+^ exclusion in stems together with leaf sheaths, thus excluding Na^+^ from leaf blades of a *japonica* rice cultivar in the reproductive growth stage, but the contribution is low when the plants are in the vegetative growth stage.

**Electronic supplementary material:**

The online version of this article (doi:10.1186/s12870-016-0709-4) contains supplementary material, which is available to authorized users.

## Background

Soil salinization causes a significant reduction in the growth and productivity of glycophytes, including major crops. In general, soil salinity is widespread in arid and semi-arid regions, particularly on irrigated land in such areas [1]. However, saline soil is also a serious problem in humid regions such as South and Southeast Asia, where encroachment of sea water occurs through estuaries and groundwater, especially in coastal regions [1]. Approximately 7 % of the total land surface suffers soil salinity to a greater or lesser extent [2]. More than 650 million hectares of land in Asia and Australia are estimated to be salt-affected, which is a serious threat to stable crop production in these densely populated areas [2].

Excessive salt accumulation triggers various detrimental effects due to two major problems: osmotic stress and ion toxicity [3–5]. Increases in osmotic pressure, caused by salt over-accumulation in the root zone, lead to a reduction in water uptake, which in turn slows down cell expansion and growth, thereby reducing cellular activity [6]. Na^+^ is a major toxic cation in salt-affected soil environments. Over-accumulated Na^+^ outside and inside of plants disturbs K^+^ homeostasis and vital metabolic reactions, such as photosynthesis, and causes the accumulation of reactive oxygen species [5, 7–9].

The high-affinity K^+^ transporter (HKT) family in plants has been extensively studied since the discovery of the *TaHKT2;1* gene from bread wheat (*Triticum aestivum*), which encodes a Na^+^-K^+^ co-transporter [10–12]. Analysis of the structure and transport properties of HKT transporters from various plant species has classified these transporter proteins into at least two subfamilies [13]. Class I HKT (HKT1) transporters were found to form a major subfamily that in general exhibits Na^+^-selective transport with poor K ^+^ permeability [4]. The single *HKT1* gene in *Arabidopsis thaliana*, AtHKT1;1, was found to be essential to cope with salinity stress [14–17]. Na^+^ channel activity mediated by AtHKT1;1 was proposed to predominantly function in xylem unloading of Na^+^ in vascular tissues, particularly in roots, which prevents Na^+^ over-accumulation in leaf blades in salt stress conditions [18–21].

In monocot crops such as rice, wheat and barley, *HKT* genes were found to form a gene family composed of genes encoding class I and class II transporters [22, 23]. QTL analyses for salt tolerance in rice plants detected a strong locus controlling K^+^ and Na^+^ contents in shoots, which was subsequently found to encode the OsHKT1;5 transporter [24]. In bread wheat, the *Kna1* locus contributing to enhanced K^+^-Na^+^ discrimination in shoots of salt-stressed plants has long been known [25, 26]. In addition, two important independent loci (*Nax1* and *Nax2*) for salt tolerance were also identified in durum wheat [27, 28]. These were shown to be responsible for maintaining low Na^+^ concentrations in leaf blades by restricting Na^+^ transport from roots to shoots [27]. It seems that the *Nax2* and *Kna1* loci are orthologs, which turned out to encode HKT1;5 transporters [29]. HKT1;5 transporters from rice and wheat plants were demonstrated to mediate Na^+^ selective transport and maintain a high K/Na ratio in leaf blades during salinity stress by preventing Na^+^ loading into xylem vessels in the roots, similar to AtHKT1;1 [24, 30, 31]. The *Nax1* locus has been shown to function in the exclusion of Na^+^ from leaf sheaths to blades in addition to restricting the movement of Na^+^ from roots to shoots [27, 32]. Sequencing analysis of the approximate mapping region of the *Nax1* locus has suggested that the effect is attributable to the *HKT1;4* gene, *TmHKT1;4*-A2 [33]. In rice, a copy of the *OsHKT1;4* gene was found in the genome [22, 23]. Recent analysis of the *OsHKT1;4* gene of a *japonica* cultivar and salt-tolerant varieties of *indica* rice suggested that the level of the *OsHKT1;4* transcript correctly spliced in leaf sheaths is closely related to the efficiency of Na^+^ exclusion from leaf blades upon salinity stress [34]. Furthermore, recent electrophysiological analyses of two TdHKT1;4 transporters from a salt-tolerant durum wheat cultivar (*Triticum turgidum*) reported Na^+^-selective transport mechanisms with distinct functional features of each transporter [35]. However, ion transport features and the physiological role of OsHKT1;4 in rice remain largely unknown.

In this study, we investigated the features of ion transport mediated by OsHKT1;4 using heterologous expression systems. We also characterized the physiological function of OsHKT1;4 under salt stress by analyzing RNAi transgenic rice lines. We found that OsHKT1;4 is a plasma-membrane (PM)-localized transporter for mediating selective Na^+^ transport, and it plays an important role in restricting Na^+^ accumulation in aerial parts, in particular, in leaf blades during salinity stress at the reproductive growth stage.

## Results

### Isolation and expression of the *OsHKT1;4* cDNA in salt hypersensitive yeast cells

To investigate the Na^+^ transport properties of OsHKT1;4, the full length *OsHKT1;4* cDNA was isolated from seedlings of the *japonica* rice cultivar Nipponbare using a specific primer set (see Methods). The isolated cDNA was 1545 bp long and deduced to encode 500 amino acids, which were completely identical to sequences registered in GenBank.

**Fig. 1:**
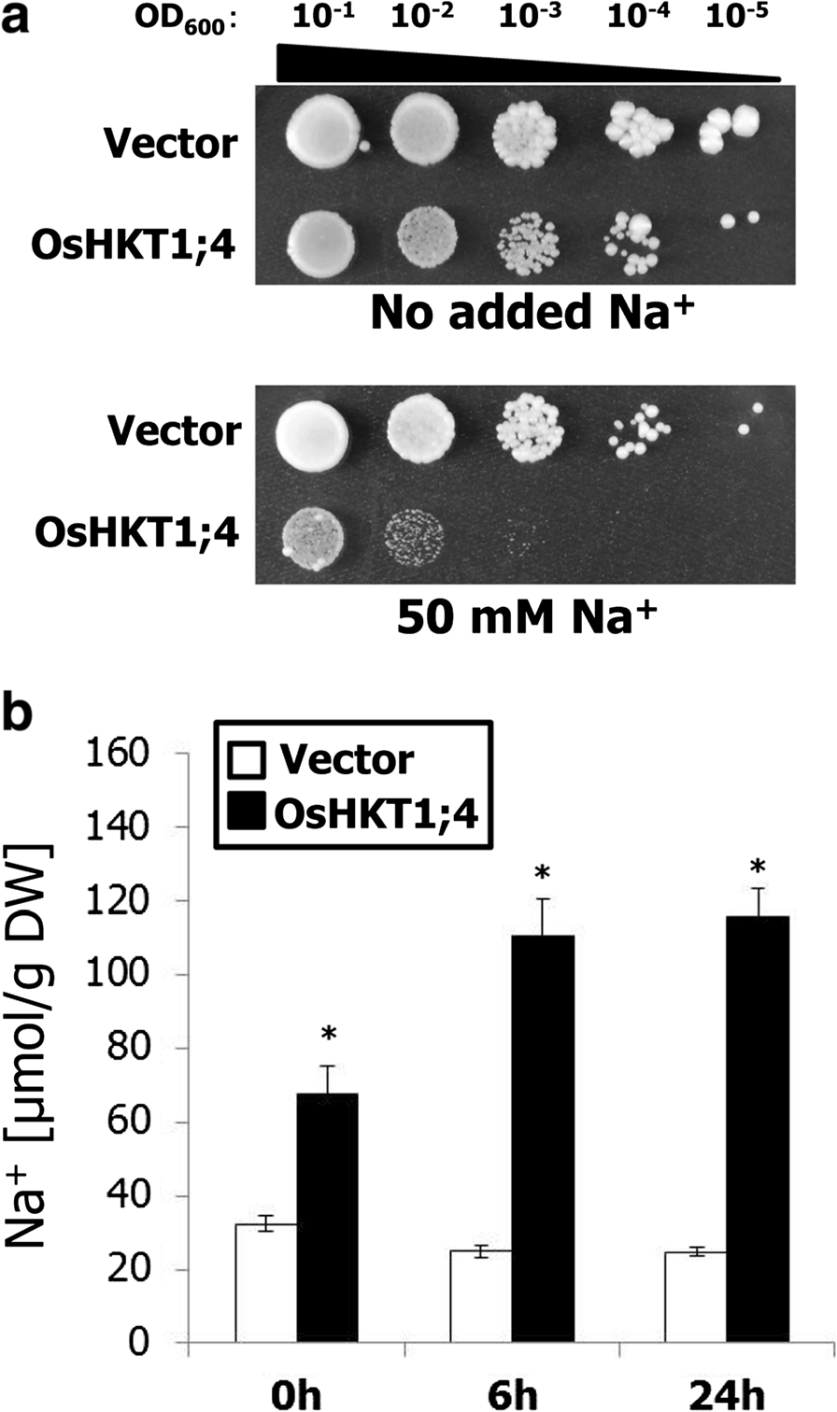
OsHKT1;4 expression increases Na^+^ hypersensitivity of yeast cells promoting Na^+^ over-accumulation. The *OsHKT1;4* cDNA from rice cultivar Nipponbare was constitutively expressed in strain G19 of *S. cerevisiae* under the control of the GAP promoter. **a**, G19 cells harboring the empty vector or expressing OsHKT1;4 were grown on the arginine phosphate (AP) medium containing 1 mM KCl with or without the addition of 50 mM NaCl. 1:10 serial dilutions of each G19 line with a starting OD_600_ of 0.1 were prepared and spotted on AP plates as described previously [51]. All plates were incubated at 30 °C, and photographs were taken after 5 days. **b**, Na^+^ content of G19 lines that were incubated in synthetic complete (SC) medium supplemented with 25 mM NaCl for the indicated time period (*n* = 6, ±SD). The Welch’s-*t* test was used for the statistical analysis and asterisks indicate a significant difference compared with vector-harboring control cells at each time point (*P* < 0.001)

Heterologous expression analysis was performed using a salt hypersensitive mutant of *S. cerevisiae* (strain G19). Transgenic G19 cells harboring an *OsHKT1;4* expression construct grew with no serious inhibition on arginine phosphate (AP) medium in the absence of excess Na^+^ although the overall growth of OsHKT1;4-expressing cells were slightly weaker than that of cells harboring empty vector (Fig. 1a). The addition of 50 mM NaCl triggered severe growth inhibition of OsHKT1;4-expressing cells in contrast to control cells on AP medium (Fig. 1a). OsHKT1;4-expressing cells accumulated significantly higher levels of Na^+^ than control cells when cultured in synthetic complete (SC) medium containing approximately 2 mM Na^+^ (Fig. 1b). Incubation with liquid SC medium supplemented with 25 mM NaCl further stimulated the phenotype, and a significant increase in Na^+^ accumulation occurred in OsHKT1;4-expressing G19 cells compared with control cells (Fig. 1b).

### Ion selectivity of OsHKT1;4 expressed in *Xenopus laevis* oocytes

**Fig. 2:**
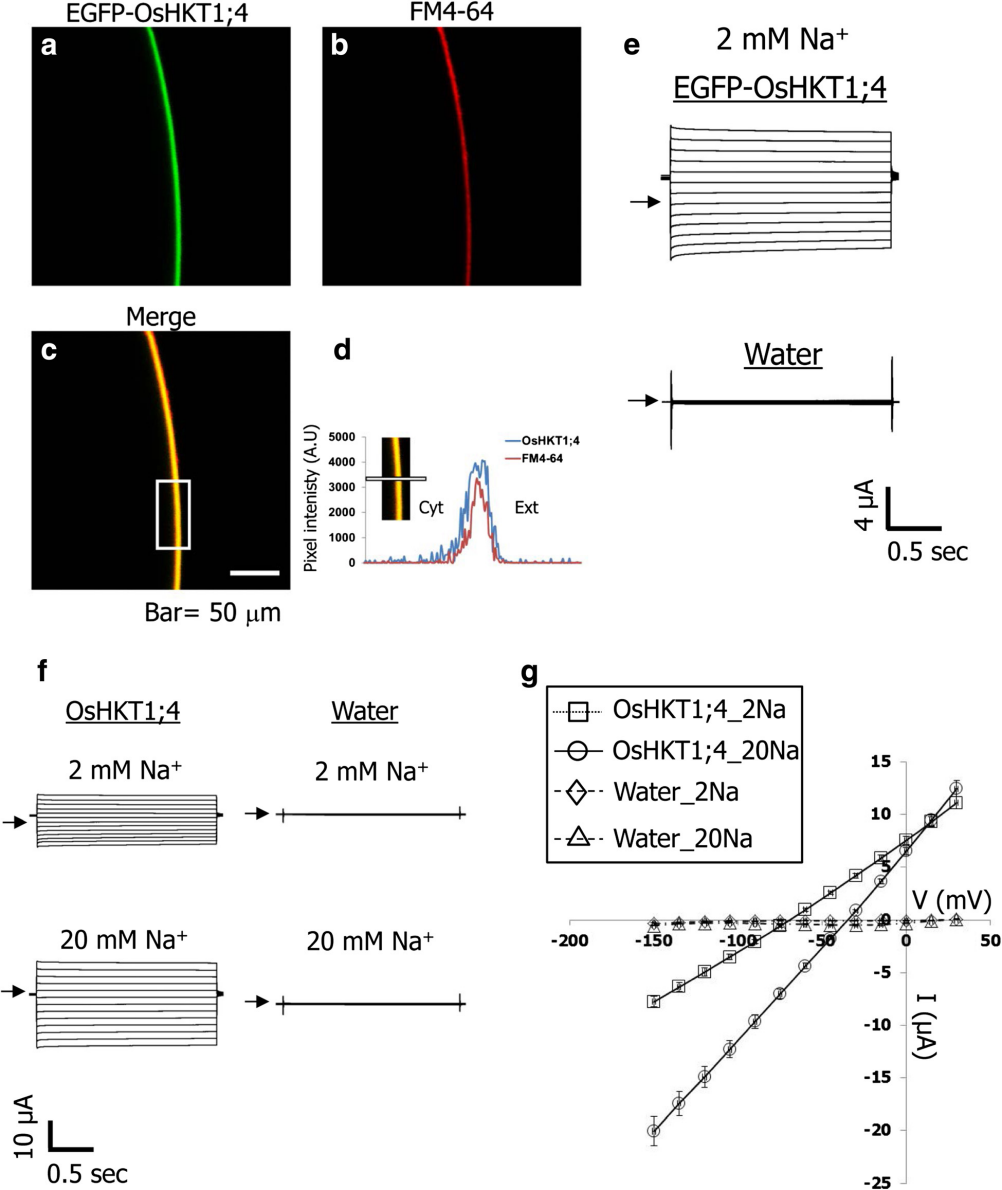
Analyses of OsHKT1;4-mediated ion transport by two electrode voltage clamp experiments using *X. laevis* oocytes. **a**, A representative confocal microscopic image of green fluorescence from oocytes injected with 3 ng of *EGFP-OsHKT1;4* cRNA. **b**, Red fluorescence of the same oocyte shown in **a**, treated with the plasma membrane marker FM4-64. **c**, Overlay image of **a** and **b**. **d**, A plot profile of EGFP (green trace) and FM4-64 (red trace) fluorescence, corresponding to the boxed region in white in panel **c** and the white line shown in the inset image. Cyt and Ext represent the cytosolic side and the external side of the plasma membrane of the oocyte, respectively. **e**, Current profiles obtained using an oocyte injected with either 3 ng of *EGFP-OsHKT1;4* cRNA (cell shown in **a**) or water in the presence of 2 mM Na^+^ with a step pulse protocol described below. Zero current levels are shown by arrows. **f**, Current profiles obtained using an oocyte injected with either 3 ng of *OsHKT1;4* cRNA or water in the presence of 2 mM or 20 mM Na^+^ with a step pulse protocol described below. **g**, Current–voltage relationships of oocytes injected with 3 ng of *OsHKT1;4* cRNA or water, bathed in solutions supplemented with 2 mM or 20 mM Na^+^ (*n* = 6-7 for *OsHKT1;4* cRNA-injected oocytes and *n* = 3-4 for water injected oocytes, ±SE). Voltage steps from +30 to −150 mV were applied with a holding potential of −40 mV

The features of OsHKT1;4-mediated ion transport were investigated by the two electrode voltage clamp (TEVC) method using *X. laevis* oocytes. To examine membrane targeting and ion transport activity of OsHKT1;4 in oocytes, a chimeric *OsHKT1;4* gene fused with the enhanced green fluorescent protein (EGFP) at the N terminus (EGFP-OsHKT1;4) was expressed. Confocal microscopy analysis indicated that EGFP-derived green fluorescence in *EGFP-OsHKT1;4* cRNA-injected oocytes overlapped with red fluorescence from the dye FM4-64, a PM marker (Fig. 2a-c). Analysis of the pixel intensity of green and red fluorescence of *EGFP-OsHKT1;4* cRNA-injected oocytes further demonstrated that EGFP-OsHKT1;4 was localized to the PM of the oocytes (Fig. 2d). In contrast, robust green fluorescence was not detected in control oocytes injected with water (data not shown). The same oocytes used for the confocal microscopic analysis were immersed in a bath containing 2 mM Na^+^ to examine whether EGFP-OsHKT1;4-dependent currents were detectable. *EGFP-OsHKT1;4* cRNA-injected oocytes showed large distinguishable currents compared with water-injected oocytes (Fig. 2e), thereby validating the functionality of the chimeric protein. We performed further TEVC experiments to characterize OsHKT1;4-mediated ion transport. Large inward and outward currents were elicited when *OsHKT1;4* cRNA-injected oocytes were bathed in both 2 mM and 20 mM Na^+^-containing solutions (Fig. 2f). Increasing the Na^+^ concentration in the bath solution led to increases in currents in OsHKT1;4-expressing oocytes, with positive shifts in the reversal potential (34.5 ± 1.3 mV), indicating the occurrence of OsHKT1;4-mediated Na^+^ transport (Fig. 2f and g). On the other hand, water-injected control oocytes showed small background currents in the same conditions (Fig. 2f and g).

**Fig. 3:**
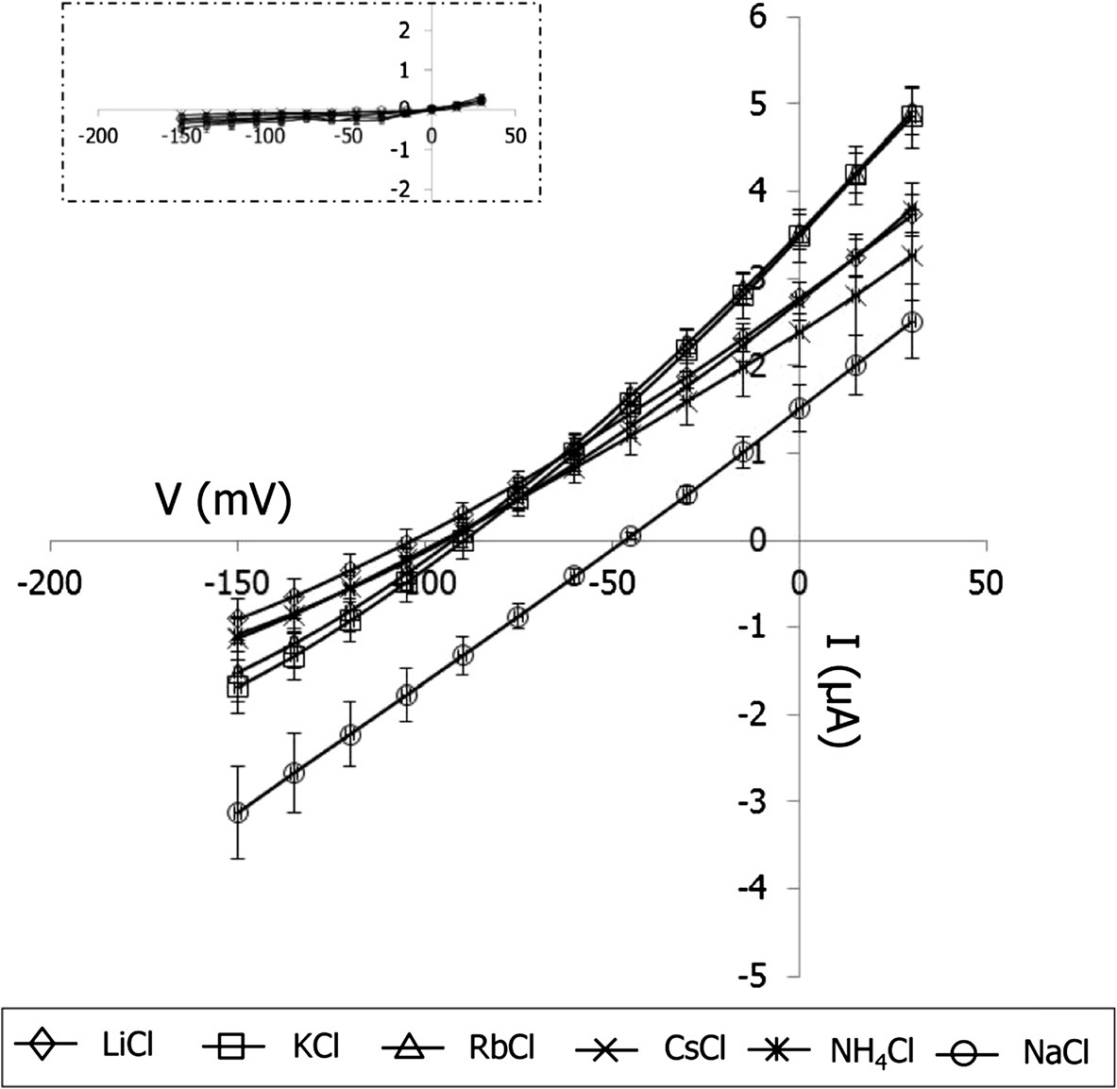
Monovalent cation selectivity of OsHKT1;4 expressed in *X. laevis* oocytes. Current–voltage relationships of oocytes injected with 3 ng of *OsHKT1;4* cRNA or water (inset), bathed in solutions containing each 10 mM chloride salt indicated in the graph (*n* = 9-11, ±SE). Voltage steps from +30 to −150 mV were applied with a holding potential of −40 mV

Monovalent cation selectivity of the OsHKT1;4 transporter was also investigated. OsHKT1;4-dependent currents were recorded by bathing *OsHKT1;4* cRNA-injected oocytes in solutions containing 10 mM cation chloride salts (Li^+^, K^+^, Rb^+^, Cs^+^, Na^+^, and NH_4_^+^). Among the six monovalent cation salts tested, 10 mM NaCl elicited the largest inward currents, with the most-positive reversal potential around −50 mV (Fig. 3). In comparison, OsHKT1;4-expressing oocytes elicited smaller and more or less similar currents in the other cation solutions, resulting in more-negative reversal potentials around −100 mV (Fig. 3).

### Subcellular localization of OsHKT1;4 in rice protoplasts

**Fig. 4:**
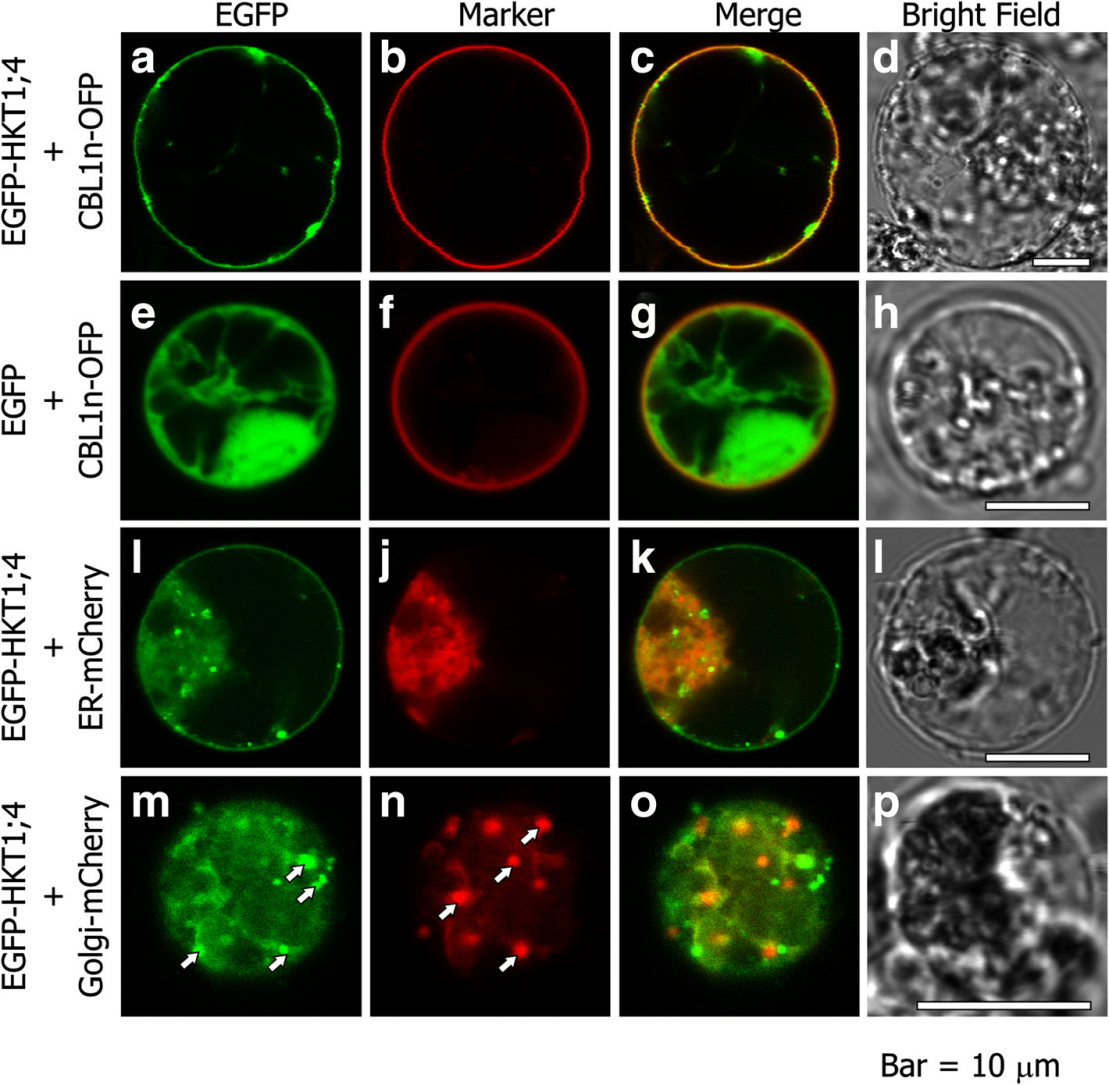
Subcellular localization of EGFP-OsHKT1;4 in rice protoplasts. EGFP-OsHKT1;4 protein was transiently expressed in protoplasts of rice seedlings under the control of the cauliflower mosaic virus 35S promoter. Fluorescence was analyzed by confocal microscopy. **a**, EGFP fluorescence (green) from a single focal plane of a representative rice protoplast co-expressing chimeric EGFP-OsHKT1;4 protein and CBL1n-OFP. **b**, OFP fluorescence (red) from the same protoplast as shown in **a. c**, Overlay image of **a** and **b. d**, Bright field image of the protoplast shown in **a. e**, EGFP fluorescence (green) from a single focal plane of a representative rice protoplast expressing free EGFP protein. **f**, OFP fluorescence from CBL1n-OFP, co-expressed in the same protoplast as shown in **e. g**, Overlay image of **e** and **f. h**, Bright field image of the protoplast shown in **e. i**, EGFP fluorescence (green) from a single focal plane of a representative rice protoplast co-expressing chimeric EGFP-OsHKT1;4 protein and ER-mCherry. **j**, mCherry fluorescence (red) marking the endoplasmic reticulum (ER) of the same protoplast as shown in **i. k**, Overlay image of **i** and **j. l**, Bright field image of the protoplast shown in **i. m**, EGFP fluorescence (green) from an internal single focal plane of a representative rice protoplast co-expressing chimeric EGFP-OsHKT1;4 protein and Golgi-mCherry (white arrows indicate punctate structures labeled by EGFP). **n**, mCherry fluorescence marking the Golgi apparatus (GA) of the same protoplast as shown in **m** (white arrows indicate typical GA structures). **o**, Overlay image of **m** and **n** showing partial co-localization of EGFP and mCherry fluorescence (corresponding to GA structures marked by arrows in N) with some punctate-like structures labeled with the EGFP alone. **p**, Bright field image of the protoplast shown in **m**

To determine the localization of the OsHKT1;4 protein in plant cells, we fused EGFP at the N-terminus end of OsHKT1;4 (EGFP-OsHKT1;4 was shown to be functional in *X. laevis* oocytes) and placed under the control of the CaMV35S promoter. Rice protoplasts transformed with EGFP-OsHKT1;4 showed the presence of EGFP fluorescence at the periphery of the cell (Fig. 4a and d). Red fluorescence from co-expressed CBL1n-OFP (orange fluorescent protein), a PM marker [36], overlapped well with the green fluorescence from EGFP-OsHKT1;4 (Fig. 4b and c). In comparison, rice protoplasts co-transformed with free EGFP and PM-marked CBL1n-OFP showed typical cytoplasmic localization of EGFP (Fig. 4e), which did not overlap with CBL1n-OFP fluorescence (Fig. 4f and g). These results strongly indicated that EGFP-OsHKT1;4 localizes to the PM of rice protoplasts. However, by repeating the transformation experiments several times, we often observed that EGFP-OsHKT1;4 was also present inside the cells and clustered in punctate-like structures (Fig. 4a, i, and m). In order to understand if the internal EGFP signal was due to the accumulation of OsHKT1;4 in the secretion pathway, we co-transformed rice protoplasts with EGFP-OsHKT1;4 together with an endoplasmic reticulum (ER) marker, ER-mCherry [37]. As shown in Fig. 4i-k, EGFP-OsHKT1;4 was present in the ER (note the yellow co-localization signal with ER-mCherry in Fig. 4k), but was also detectable at the PM (Fig. 4i and k), which was not labeled with mCherry (Fig. 4j). This latter result indicated that EGFP-OsHKT1;4 was partially retained in the ER, but that it was also able to properly reach the PM. Moreover, co-expression of EGFP-OsHKT1;4 with the ER marker revealed that the observed EGFP punctate-like structures were not made of ER membranes, because they did not exhibit mCherry fluorescence. We further investigated if such punctate-like structures could be a part of the Golgi apparatus (GA) by co-expressing EGFP-OsHKT1;4 with a Golgi marker, Golgi-mCherry [37] and analyzing optical sections of transformed protoplasts in which the GA was clearly detectable (Fig. 4m-o). As shown in Fig. 4o, EGFP and mCherry fluorescence only partially overlapped (yellow signal), with some punctate-like structures that were labeled with EGFP alone (arrows in Fig. 4m). This latter result indicated that EGFP-OsHKT1;4 was also present in the GA as well as in still unidentified structures.

### Expression profiles of the *OsHKT1;4* gene in a *japonica* cultivar of rice

**Fig. 5:**
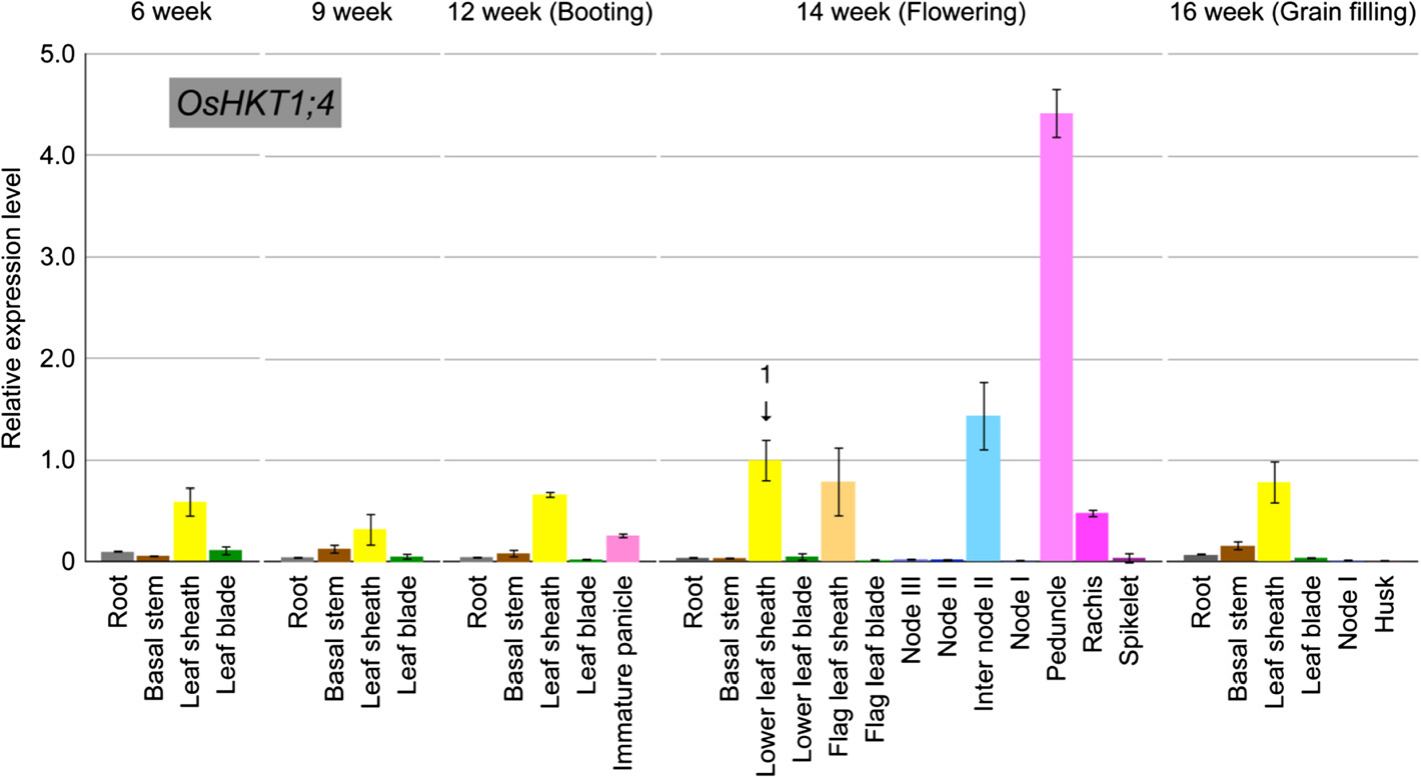
Growth stage-dependent expression of *OsHKT1;4* in various tissues of a *japonica* rice cultivar Nipponbare. RNA samples from various tissues were prepared from rice plants at the indicated growth stages as described previously [38], and quantitative real-time PCR analysis was performed using specific primers for *OsHKT1;4* (*n* = 3, ±SD). Relative expression of *OsHKT1;4* is shown, with its relative expression in the lower leaf sheath of 14-week-old (flowering) plants to 1

We investigated the tissue-specific expression pattern of *OsHKT1;4* at various growth stages of rice plants using the same samples reported previously [38]. Higher expression of *OsHKT1;4* in leaf sheaths was found throughout the growth periods (Fig. 5). At the flowering stage, the highest expression level was found in the peduncle and internode II (Fig. 5). Note that lower levels of *OsHKT1;4* expression were also detected in other organs (Fig. 5).

**Fig. 6:**
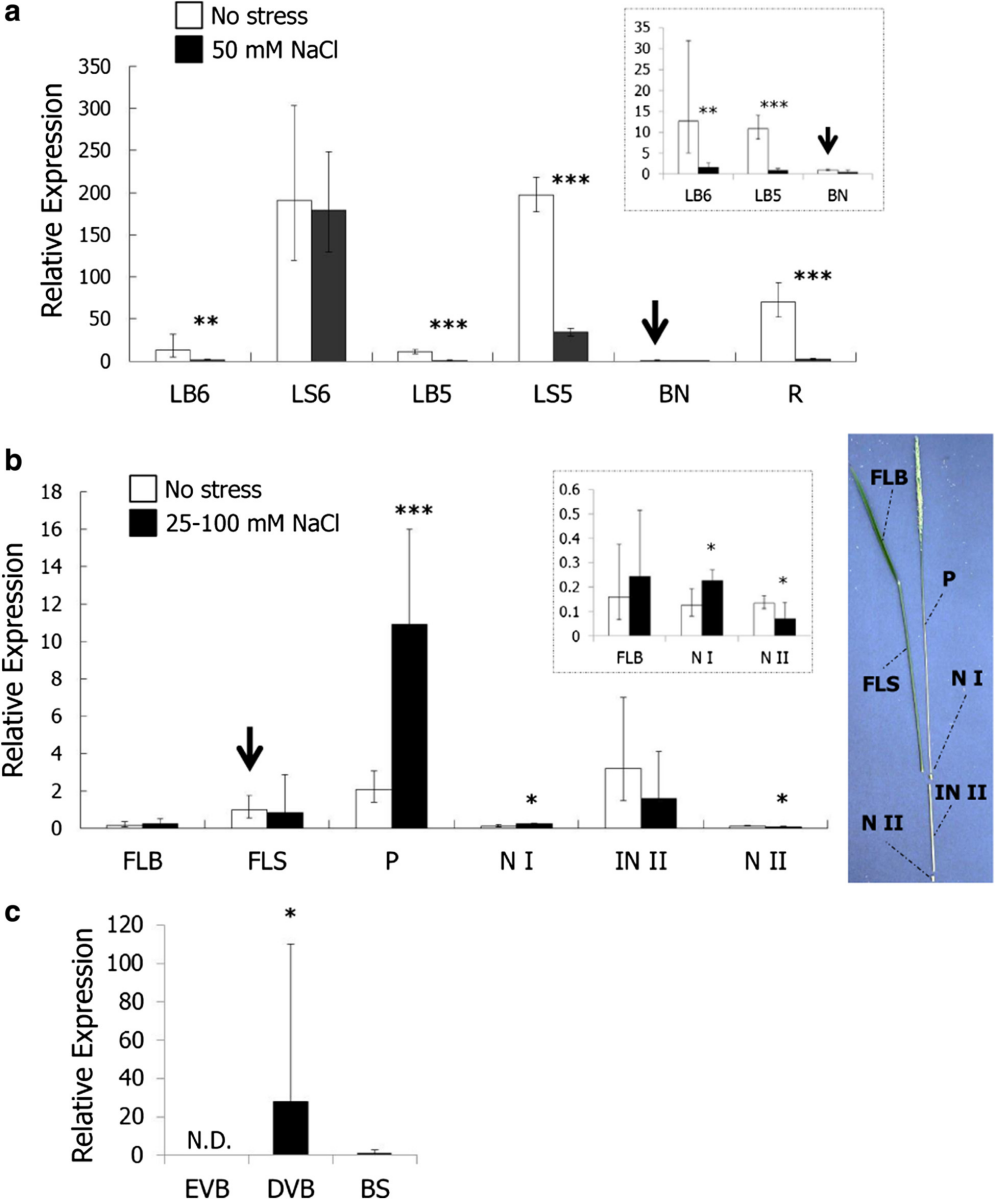
Expression profile of *OsHKT1;4* in various tissues of Nipponbare rice plants. Quantitative real-time PCR analyses were performed using RNA samples derived from various tissues. The expression of *OsHKT1;4* and an internal control *OsSMT3* was determined. The level of *OsHKT1;4* expression was normalized using *OsSMT3* expression. **a**, Relative expression of *OsHKT1;4* in tissues of hydroponically grown Nipponbare plants in the vegetative growth phase (3-weeks-old) with or without 50 mM NaCl treatments for 3 days is shown setting its expression in the basal node without stress to 1 (*n* = 6, ±SD). LB6: 6th leaf blade; LB5: 5th leaf blade; LS6: 6th leaf sheath; LS5: 5th leaf sheath; BN: basal node; R: root. **b**, Relative expression of *OsHKT1;4* in tissues of soil-grown Nipponbare plants in the reproductive growth phase with or without NaCl treatments (25–100 mM) for more than 1 month is shown setting its expression in the flag leaf sheath to 1 (*n* = 5-6, ±SD). FLB: flag leaf blade; FLC: flag leaf sheath; P: peduncle; N I: node I; IN II: internode II; N II: node II. Note that insets in **a** and **b** show the data sets from some tissues in a smaller scale than that in the main graphs. **c**, Relative *OsHKT1;4* expression in enlarged vascular bundles (EBVs) of node I, diffuse vascular bundles (DVBs) of node I, and the basal stem (BS) is shown setting its expression in the basal stem to 1 (*n* = 6, ±SD). EVBs and DVBs were excised from node I by laser microdissection (LMD). N.D. indicates “not detected”. The Welch’s-*t* test was used for the statistical analysis: * *P* < 0.05, ** *P* < 0.01, *** *P* < 0.001 vs. no stress condition (**a**, **b**) or basal stem (**c**)

We further investigated the response of *OsHKT1;4* to stress at two different growth stages. At the vegetative growth stage, exposure to 50 mM NaCl resulted in significant reductions in the accumulation of *OsHKT1;4* transcripts in all organs except the youngest leaf sheath (Fig. 6a, LS6). A stepwise 25 mM increase in the NaCl concentration every 3 days from 75 mM to 100 mM was subsequently applied to 50 mM NaCl-treated plants, and the same organs were harvested at each NaCl concentration. In general, prolonged and increased NaCl stress maintained severe reductions of *OsHKT1;4* expression in young leaf blades, leaf sheaths, basal nodes and roots compared with control plants (Additional file 1: Figure S1). One characteristic difference from 50 mM NaCl-treated plants was the expression profile in the youngest leaf sheath (the 7th leaf sheath with 75 mM NaCl and the 8th leaf sheath with 100 mM NaCl, respectively), in which *OsHKT1;4* expression showed significant reductions as in other tissues, and the decrease-trend became more severe as the strength of NaCl stress increased (Additional file 1: Figure S1).

At the reproductive stage, *OsHKT1;4* transcript levels were significantly increased in peduncles in response to salt stress (Fig. 6b, P). In addition, a significant increase in *OsHKT1;4* expression was also found in the uppermost node of salt-stressed rice plants compared with control plants, although the basal level of *OsHKT1;4* expression in the tissue was relatively low (Fig. 6b, inset, N I).

The node is an essential tissue for distributing minerals, and toxic elements, that are transported from the roots [39]. The node includes different types of vascular bundles (VBs) such as enlarged VBs (EVBs) and diffuse VBs (DVBs), each of which have distinct functions in the distribution of elements [39]. Given that the level of expression of *OsHKT1;4* was elevated in node I in response to salinity stress (Fig. 6b), we examined the expression pattern of *OsHKT1;4* in EVBs and DVBs by combinational analysis of laser microdissection (LMD) and real-time PCR. As shown in Fig. 6c, *OsHKT1;4* expression was predominantly detected in DVBs but not EVBs in node I, which was approximately 28-times higher than the expression in the basal stem (Fig. 6c).

### Phenotypic analysis of *OsHKT1;4* RNAi plants in salinity stress conditions

To investigate whether OsHKT1;4-mediated Na^+^ transport contributes to salt tolerance in rice plants, we generated *OsHKT1;4* RNAi plants. Two independent transgenic lines, which showed reductions in *OsHKT1;4* expression in leaf sheaths during the reproductive growth phase, were selected and used for phenotypic analysis (Additional file 2: Figure S2A). Growth with 50 mM NaCl in hydroponic culture for more than 2 weeks in Nipponbare and RNAi lines did not cause any difference in visual characteristics (data not shown). The Na^+^ concentration of different organs was compared between WT and RNAi plants after the plants were treated with 50 mM NaCl for 3 days. No difference was found in the Na^+^ concentration of all organs between WT and RNAi lines (Additional file 2: Figure S2B).

**Fig. 7:**
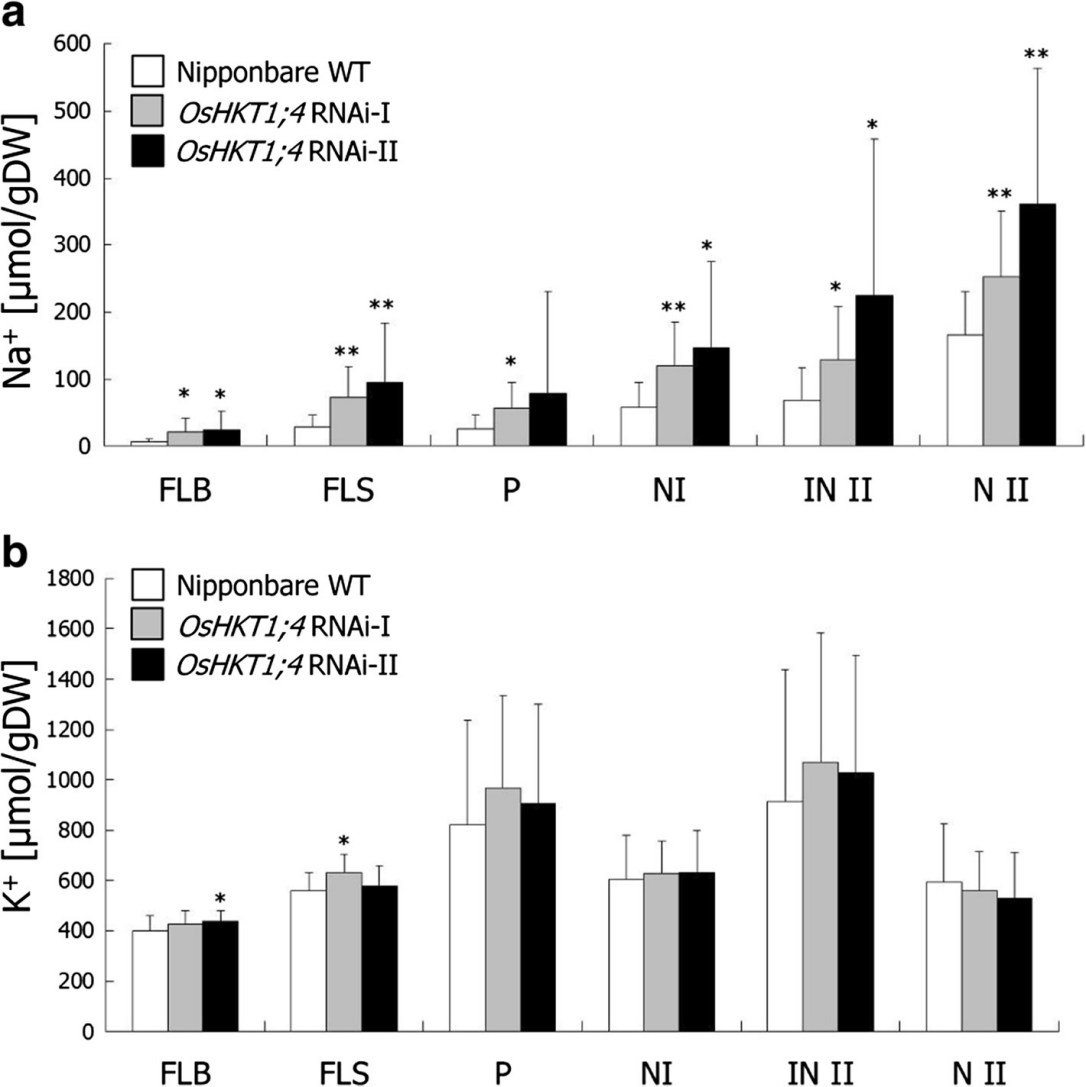
Phenotypic analysis of *OsHKT1;4* RNAi plants in the reproductive growth stage. Nipponbare wild-type and two independent *OsHKT1;4* RNAi plants were planted in the same pot filled with paddy-filed soil and grown for approximately 3 months. When the plants started heading, NaCl treatment was initiated by gradually increasing the concentration of Na^+^ in tap water from 25 mM to 100 mM for more than a month. Tissues of the upper parts were excised and washed briefly by the ultrapure water. Ion contents were determined using an inductively coupled plasma optical emission spectrometer (*n* = 23-28, ±SD). **a**, Na^+^ content in each tissue. **b**, K^+^ content in each tissue. FLB: flag leaf blade; FLC: flag leaf sheath; P: peduncle; N I: node I; IN II: internode II; N II: node II. The Welch’s-*t* test was used for the statistical analysis: * *P* < 0.01, ** *P* < 0.001 vs. Nipponbare wild-type

Given that *OsHKT1;4* expression in the tissues of rice at the vegetative growth stage was down-regulated, but was up-regulated in some tissues at the reproductive growth stage in response to NaCl stress (Fig. 6a and b), we then examined the phenotypes of RNAi lines at the reproductive growth stage in high-salinity conditions. Wild-type Nipponbare plants and each *OsHKT1;4* RNAi line were planted in the same pot filled with soil from paddy fields and grown in two independent greenhouse facilities at two different institutes. Nipponbare and *OsHKT1;4* RNAi plants were watered with tap water containing 25 mM NaCl when they started heading, and the NaCl concentration was gradually elevated with a 25 mM increase to the maximum concentration of 100 mM for more than a month. Flag leaves, peduncles, nodes (I and II) and internode IIs were harvested and ion contents were determined. Generally, knockdown of *OsHKT1;4* resulted in the accumulating more Na^+^ in every organ tested (Fig. 7a). In particular, the largest influence from the reduction in *OsHKT1;4* expression was observed in the Na^+^ content of flag leaf blades, where approximately 3.5–4-fold increases in Na^+^ content in RNAi plants was detected on average compared with that of wild-type plants (Fig. 7a). Flag leaf sheaths of the RNAi plants also exhibited 2.5–3-fold increases in Na^+^ accumulation compared with control plants (Fig. 7a). In contrast, no marked difference in K^+^ accumulation between the wild-type and RNAi plants was found in all organs tested (Fig. 7b).

After the completion of NaCl stress treatment with the soil-grown rice plants, a proportion of the plants were subsequently maintained by watering with normal tap water to investigate the Na^+^ content in the mature rice grains. The Na^+^ content of ripening grains was 25–34 % higher in RNAi plants compared with wild-type plants (Additional file 3: Figure S3A). In contrast, the Na^+^ contents of non-ripening grains and rachis-branches tended to be highly variable, but no noticeable difference was observed between wild-type and *OsHKT1;4* RNAi plants (Additional file 3: Figure S3B).

We also conducted a ^22^Na^+^-tracer analysis on *OsHKT1;4* RNAi and Nipponbare WT plants. The inflorescence including the peduncle and ear excised from the edge of node I was soaked in a solution containing ^22^Na^+^ for the direct absorption. As a result, peduncles from *OsHKT1;4* RNAi plants tended to allow the transfer of a larger amount of Na^+^ from the cut-end to the upper regions in comparison with WT (Additional file 4: Figure S4) with an exception of an independent plant from the *OsHKT1;4* RNAi-II line, which exhibited a similar tracer profile to that of WT (Additional file 4: Figure S4G).

## Discussion

### Ion transport properties of OsHKT1;4 expressed in heterologous cells

Na^+^-selective transport mediated by some class I HKT (HKT1) transporters have been indicated to play a crucial role in Na^+^ exclusion from leaves of salt-stressed plants [16, 19, 20, 24, 29–31, 33, 40]. The *HKT1;4*-A2 locus in durum wheat, which was derived from a wild wheat relative *Triticum monococcum*, was highlighted as a strong candidate for a salt tolerance QTL named *Nax1* [27, 28, 33]. In rice, a role of OsHKT1;4 in controlling Na^+^ concentrations in leaf blades was suggested by comparative analyses of Na^+^ contents in leaf blades and the level of *OsHKT1;4* transcripts in sheaths using salt-tolerant *indica* rice varieties and a *japonica* rice cultivar Nipponbare [34]. However, the ion transport properties and physiological functions of OsHKT1;4 remain to be elucidated.

Stable and constitutive expression of OsHKT1;4 in a salt hypersensitive strain of *S. cerevisiae* G19 led to an increase in sensitivity to increases in extracellular NaCl concentration, with significant increases in Na^+^ accumulation in the cells (Fig. 1). Plasma membrane-targeted OsHKT1;4 was found to elicit large currents stimulated by Na^+^ in *X. laevis* oocytes with shifts in zero-current potentials toward a more depolarized status, dependent on increases in the Na^+^ concentration in the bath solution (Fig. 2a-g). A 10-fold increase in the Na^+^ concentration in the bath resulted in the shift of the reversal potential of 34.5 ± 1.3 mV on average (with the reversal potentials of −69.3 ± 3.3 mV and −34.8 ± 0.7 mV in the 2 mM and 20 mM Na^+^ solutions, respectively; Fig. 2g), which was smaller than the theoretical Nernstian shift of 58–59 mV. Note however that the reversal potential shifts of OsHKT1;4-expressing oocytes can be less than the theoretical value because the cytoplasmic Na^+^ concentrations of the oocytes may also shift due to the function of OsHKT1;4. Further experiments will be needed to characterize the ion selectivity of OsHKT1;4 in detail. In addition, investigation of monovalent cation selectivity of OsHKT1;4 expressed in oocytes bathed in solutions containing solo cation-chloride salts further revealed that this transporter is highly selective for Na^+^ amongst Li^+^, K^+^, Rb^+^, Cs^+^, Na^+^, and NH_4_^+^ (Fig. 3). These results indicate that OsHKT1;4 is a Na^+^ transporter.

HKT proteins have been suggested to contain four selectivity-filter-pore (p-loop) domains that are distantly related to a bacterial K^+^ channel [41–44]. HKT1 transporters have been found to be highly selective for Na^+^, and in general a serine residue at the key amino acid position for K^+^ selectivity in the first p-loop domain is conserved instead of a glycine residue, which corresponds to the first glycine in the GYG motif of the shaker-type K^+^ channel [10]. Corresponding amino acid positions in the three other p-loop domains of OsHKT1;4 were reported to be glycine residues, resulting in a SGGG type for the p-loop domains of OsHKT1;4 as typical HKT1 transporters [10]. The property of Na^+^ selective transport by OsHKT1;4 was consistent with the prediction of Na^+^ selectivity of HKT transporters based on the p-loop hypothesis [10, 41, 42].

### The role of OsHKT1;4 in salt tolerance mechanisms in rice

QTL analyses for salt tolerance of durum wheat plants have led to the identification of the salt tolerance-determining *Nax1* locus, which was deduced to be the *TmHKT1;4-A2* gene [27, 28, 33]. The *Nax1* locus-mediated xylem Na^+^ unloading in roots and leaf sheaths of durum wheat plants has been suggested to avoid Na^+^ over-accumulation in leaf blades during salinity stress [27]. Relatively steady expression in leaf sheaths throughout growth stages is a distinctive feature of the *OsHKT1;4* gene in Nipponbare plants (Figs. 5, 6a and b). In 3-week-old Nipponbare plants, grown in hydroponic culture, the expression of *OsHKT1;4* was also observed in roots (Fig. 6a). However, the level of *OsHKT1;4* expression mostly showed significant decreases in tissues/organs of salt-stressed Nipponbare plants at the vegetative growth stage under salinity stress (Fig. 6a, Additional file 1: Figure S1). *OsHKT1;4* RNAi plants in the vegetative growth stage did not show any noticeable difference either in visual phenotype or in Na^+^ content after the imposition of 50 mM NaCl stress compared with Nipponbare wild-type plants (Additional file 2: Figure S2). These results suggested a possibility that OsHKT1;4-mediated Na^+^ transport does not provide a profound contribution to vital Na^+^ homeostasis during the vegetative growth phase of the *japonica* rice cultivar during salinity stress.

Another characteristic feature of *OsHKT1;4* gene expression was its high expression in the stem, including the peduncle and the internode II, of rice plants at the reproductive growth stage (Fig. 5). The observed expression profile of *OsHKT1;4* was consistent with that found in the RiceXPro database, in which *OsHKT1;4* is highly up-regulated in the stem of rice plants in heading and ripening stages (http://ricexpro.dna.affrc.go.jp/) [45]. Long-term salinity stress treatment with gradual increases in NaCl concentration in soil-grown Nipponbare plants from heading to ripening stages led to significant increases in *OsHKT1;4* expression in the peduncle, with relatively steady expression levels in the flag leaf blade and internode II independent of salt treatments (Fig. 6b). Significant up-regulation of *OsHKT1;4* expression was also observed in node I, although the basal expression level in this tissue was far less than that in the peduncle (Fig. 6b). Similar long-term salinity stress treatments of soil-grown *OsHKT1;4* RNAi and wild-type plants resulted in significantly higher Na^+^ contents in aerial tissues of RNAi plants, with the highest impact on the Na^+^ content of flag leaf blades compared with wild-type plants (Fig. 7a). Together with Na^+^ selective transport mediated by plasma membrane-targeted OsHKT1;4 (Figs. 1, 2, 3 and 4), these results suggested that OsHKT1;4 contributes to the prevention of Na^+^ over-accumulation in aerial parts, in particular leaf blades of Nipponbare plants that are in the reproductive growth stage, during salinity stress. HKT1;4 transporters in wheat have been suggested to function in xylem Na^+^ unloading in roots and leaf sheaths upon salinity stress to reduce Na^+^ transfer into leaf blades [27]. On the other hand, HKT1-mediated Na^+^ recirculation via the downward stream of the phloem has been argued as a potential working model for HKT1 transporters [4, 14, 20, 27]. *OsHKT1;4* RNAi plants in the reproductive growth stage accumulated more Na^+^ not only in leaves but also in tissues of the stem investigated under salinity stress (Fig. 7a). The reason for the phenotype is not clear yet. In a previous study, analyses on *athkt1;1* mutants of Arabidopsis indicated that the dysfunction of AtHKT1;1-mediated Na^+^ unloading from xylem caused the impairment of Na^+^ recirculation via phloem as well, which could together be attributed to Na^+^ over-accumulation in shoots of *athkt1;1* mutants upon salinity stress [20]. ^22^Na^+^-imaging analysis indicated a higher amount of the Na^+^ transfer in peduncles of *OsHKT1;4* RNAi plants than WT plants with an exception of an independent *OsHKT1;4* RNAi-II plant (Additional file 4: Figure S4), suggesting that OsHKT1;4 mediates Na^+^ unloading from xylem and reduced activity of OsHKT1;4 leads to an increase in Na^+^ accumulation in this tissue. Together, Na^+^ over-accumulation in aerial parts of *OsHKT1;4* RNAi plants upon salinity might be due to the insufficient activity of OsHKT1;4 in Na^+^ unloading from xylem, which in turn could also bring about inhibition of Na^+^ recirculation. To elucidate the precise function of OsHKT1;4 in Na^+^ exclusion in rice, a detailed investigation into whether OsHKT1;4 predominantly mediates xylem Na^+^ unloading or phloem-involved Na^+^ recirculation or both will be an essential question to be addressed in future research. In this respect, it will be also interesting to study the *in planta* localization of OsHKT1;4 in order to investigate possible regulatory mechanisms that can control its PM localization, and hence Na^+^ transport and salt tolerance. Indeed, our subcellular localization analyses leave room for some speculation. Using rice protoplasts, we could clearly observe the PM localization of OsHKT1;4 (in accordance with *X. laevis* oocytes data), but also the presence of the protein in unidentified vesicles that were neither ER nor GA structures (Fig. 4). This vesicle could represent a means (e.g. through endosomes) to control the abundance of OsHKT1;4 in the PM. Recently it has been demonstrated that another member of the HKT family, OsHKT1;3, is targeted to the GA and undergoes strict control of its trafficking [46]. Note that the subcellular localization of OsHKT1;4 was investigated using rice protoplasts over-expressing the chimeric EGFP-OsHKT1;4 protein. The hypothesis that endocytotic mechanisms regulate the amount of OsHKT1;4 in the PM requires further investigation using rice plants.

In addition to aerial tissues, increases in the Na^+^ content of ripening grains from *OsHKT1;4* RNAi plants were found during salinity stress conditions compared with wild-type plants (Additional file 3: Figure S3A). The expression of *OsHKT1;4* was up-regulated by salt stress in the peduncle and node I (Fig. 7b). In Node I, it was also found by LMD-combined qPCR analysis that the *OsHKT1;4* gene was predominantly expressed in the DVB, which is connected to the ear of rice (Fig. 7c) [39]. Taken together, these results suggested a potential contribution of OsHKT1;4 in protecting reproductive organs and seeds from Na^+^ toxicity in rice plants in addition to the leaf blades upon salinity stress (Figs. 6b and c, Additional file 3: Figure S3A).

## Conclusions

In this study, we have characterized the ion transport properties of OsHKT1;4 and investigated its impact on Na^+^ homeostasis during salinity stress. Our results revealed that OsHKT1;4-mediated Na^+^ selective transport does not have a significant influence on Na^+^ accumulation in major tissues/organs of a *japonica* rice cultivar during the vegetative growth stage in salt-stress conditions (Additional file 2: Figure S2). However, we found a non-negligible impact of the function of OsHKT1;4 on Na^+^ accumulation in aerial parts at the reproductive growth stage (Fig. 7a, Additional files 3 and 4: Figures S3A and 4). Recently, mutations in a key transcription factor controlling *OsHKT1;1* conferred significant salt tolerance to rice plants, suggesting a major impact of OsHKT1;1 in the salinity tolerance of rice [47]. Consistently, a null mutation in the *OsHKT1;1* gene of Nipponbare plants has been recently demonstrated to render the plants salt hypersensitive [40]. Together with evidence for the essential role of the OsHKT1;5 transporter in salt tolerance [24], these findings suggest a possibility that multiple OsHKT1 transporters mediate the mechanism of Na^+^ exclusion from aerial parts including leaf blades in rice plants. To fully understand the role of OsHKT1;4-mediated Na^+^-selective transport on the salt-resistance mechanism and the yield of salt-stressed rice plants, elucidation of the tissue specificity of OsHKT1;4 and the analysis of rice mutants that harbor null mutations in the *OsHKT1;4* gene will be crucial.

## Methods

### Plant material and growth condition

A *japonica* rice cultivar Nipponbare (*Oryza sativa* L.) was used as a standard wild-type in this study. Seeds were surface sterilized and germinated as described previously [48]. For the preparation of rice plants in the vegetative growth stage, seedlings were transferred to plastic pots containing half-strength Kimura B nutrient solution [49]. Hydroponic culture was performed in a light/dark cycle of 16/8 h and a temperature regimen of 30/28 °C using a growth chamber (FLI-301NH; EYELA, Tokyo, Japan) and the solution was changed every 3 days. Saline hydroponic solution containing 50 mM NaCl was applied with approximately 3-week-old plants for 3 days. A stepwise 25 mM increase in NaCl concentration in the hydroponic solution was performed every 3 days as necessary (in total 6 days for 50 to 75 mM and 9 days for 50 to 75 to 100 mM treatments).

For the preparation of rice plants in the reproductive growth stage, seedlings were transferred to plastic pots filled with the paddy-field soil and grown in greenhouses. Two independent greenhouse facilities at two different institutes were utilized for this experiment. When the plants started heading, salt-stress treatments were initiated with 1.5 L of tap water containing 25 mM NaCl. A gradual 25 mM increase in NaCl concentration in 1.5 L of tap water was performed as follows: once for 50 mM NaCl, three times for 75 mM NaCl, and twice for 100 mM NaCl. Plants were watered normally afterwards until grain harvest.

As for the growth stage-dependent expression analysis, rice plants were prepared as described [38]. In brief, 3-week-old Nipponbare WT plants were prepared by hydroponic culture and then transplanted to the paddy field. Indicated samples were taken at both vegetative and reproductive growth stages.

### Isolation of *OsHKT1;4* cDNA and constructs for heterologous expression analyses

The *OsHKT1;4* cDNA was isolated from rice seedlings using specific primers: Forward, 5’-TGCTCCAATATGCCCACGTC-3’, Reverse, 5’-CCTGCAATGTTCAGCTGGTACTG-3’. The isolated cDNA was amplified by PCR using specific primers containing *Xba*I (5’) and *Bam*HI (3’) restriction sites: Forward, 5’-ATCTAGACATGCCCACGTCGCGGC-3’, Reverse, 5’-TGGATCCCTAACTAAGTTTCCAGGCTTTGCCT-3’. The amplified fragments were subcloned downstream of EGFP in frame in pBI221 for transient expression in rice protoplasts [50]. For expression in *X. laevis* oocytes, the entire EGFP-OsHKT1;4 sequence was PCR amplified using *Bgl*II site-containing primers for subcloning into pXßG-ev1 [48]. The primer sequences were: Forward, 5’-CATGAGATCTATGGTGAGCAAGGGCGAG-3’, Reverse, 5’-CATGAGATCTCTAACTAAGTTTCCAGGCTTTGCCT-3’. All DNA constructs used in this study were checked by DNA sequencing.

### Preparation of *OsHKT1;4* knockdown lines

To produce *OsHKT1;4* RNAi knockdown lines of Nipponbare plants, an *OsHKT1;4* RNAi construct was prepared as follows: a 419 bp region of the *OsHKT1;4* cDNA was amplified by PCR using primer sets: Forward, 5’-ATGTCCACCGTCGAGATGGA-3’ attached with either a *Bam*HI or an *Eco*RV site, and Reverse 5’-GTCCACGTGTTCAGCGACTTGT-3’ attached with either an *Xba* I or a *Bgl*II site. Two copies of the fragment were subcloned into p2K-1^+^ [50] and located under the control of the maize ubiquitin 1 promoter as inverted repeats sandwiched between a GUS-loop region. Transgenic rice (cv. Nipponbare) was produced using *Agrobacterium tumefaciens*-mediated transformation. The *OsHKT1;4* expression levels in the leaf sheaths of 11 independent transgenic candidates in the reproductive growth phase were surveyed by real-time PCR and two independent lines were selected and used in this study.

### OsHKT1;4 expression in *S. cerevisiae*

The *OsHKT1;4* cDNA was subcloned into pRS406 harboring promoter and terminator regions of the glyceraldehyde-3-phosphate dehydrogenase (GAP) gene. *Saccharomyces cerevisiae* strain G19 that was disrupted in genes encoding a Na^+^-ATPase was used for salt sensitivity growth tests, as described previously [48, 51]. Selected transformants were assayed using AP medium supplemented with 1 mM KCl with or without 50 mM NaCl. Pre-cultured vector-containing control and *OsHKT1;4* cDNA-containing cells were washed with AP medium with no additional K^+^ and Na^+^, and 1:10 serial dilutions were subsequently prepared with a maximum OD_600_ of 0.1. These were spotted onto each AP plate, and all plates were incubated at 30 °C for 5 days.

### Electrophysiology using *X. laevis* oocytes

The *OsHKT1;4* cDNA was subcloned into pXßG-ev1, and cRNA was transcribed using a mMESSAGE mMACHINE *in vitro* transcription kit (Ambion®Life Technologies, Japan). Oocytes were prepared, and TEVC experiments were performed as described previously [52] with a minor modification. In brief, 3 ng of cRNA (*OsHKT1;4*, *EGFP-OsHKT1;4*) was injected into *X. laevis* oocytes and incubated at 18 °C for 1 day. Water-injected oocytes were also prepared as controls at each experiment. TEVC recordings and data analysis were performed using an Axoclamp 900A amplifier (Molecular Devices, USA) and an Axon Instruments Digidata 1440A and pCLAMP 10 (Molecular Devices). As for the analyses of ion selectivity using alkali cation salts, oocytes were bathed in a solution containing, 6 mM MgCl_2_, 1.8 mM CaCl_2_, 10 mM MES-1,3-bis (tris[hydroxymethyl]methylamino) propane, 180 mM D-mannitol (pH 5.5) (with BisTrisPropane), and the indicated concentrations of Na glutamate salts or cation chloride salts. The osmolality of each solution was adjusted to 230 to 250 mosmol kg^−1^ with D-mannitol. Voltage steps were applied from +30 to −120 or −150 mV in 15-mV decrements, with a holding potential of −40 mV. All experiments were performed at room temperature.

TEVC experiments were performed in Okayama University and UC San Diego. No ethics approval was required for the study using *Xenopus* frogs in Okayama University based on the article 2 in the Policy on the Care and Use of the Laboratory Animals, Okayama University (H200221-6). Research at UCSD was permitted by a UCSD tissue transfer permit (T13284) for use of oocytes.

### Real-time PCR analysis

Total RNA was extracted using an RNeasy Plant Mini Kit (Qiagen, Limburg, Netherlands), and reverse transcription was performed using PrimeScript™ RT Master Mix in accordance with the manufacturer’s protocol (Takara, Japan). A Thermal Cycler Dice Real Time System II TP800 (Takara) was used for the real-time PCR analysis. The Q-PCR analysis was performed setting the maximum cycle number of 40 in accordance with the protocol of the manufacturer (Takara, Japan). *OsSMT3*, *HistoneH3* or *Actin* genes were used as internal controls as described previously [50]. Gene-specific primers for *OsHKT1;4* were used: Forward, 5’-GTCGAAGTTGTCAGTGCATATGG-3’; Reverse, 5’-TGAGCCTCCCAAAGAACATCAC-3’.

To analyze the tissue specific expression of *OsHKT1;4* in node I, regions of the enlarged vascular bundle (EVB) and the diffuse vascular bundle (DVB) were excised by the laser microdissection (LMD: Arcturus Laser Capture Microdissection System, Life Technologies, CA, USA) as described previously [53].

### Subcellular localization analysis using protoplasts from leaf sheaths of rice seedlings

Seven-day-old rice seedlings were used for the isolation of leaf sheath protoplasts. The protoplasts were isolated and transformed as described previously [54]. The amounts of DNA used for the transformation were: 2 μg for EGFP-OsHKT1;4; 1 μg for CBL1n-OFP (PM marker: [36]; 2 μg of nWAK2-mCherry-HDEL (ER marker: [37]; 2 μg for Man49-GFP (GA marker: [37, 55] and 1 μg of free EGFP [51]. For the co-transformation experiments, the DNA constructs were mixed together before adding them to isolated rice protoplasts, and then polyethylene glycol (PEG 4000) solution was added and mixed gently. After removing the PEG, the protoplasts were re-suspended in WI solution (0.5 M mannitol, 20 mM KCl, 4 mM MES, pH 5.7) and kept in the dark at 24 °C for 12–16 h before microscopy analysis.

Confocal microscope analyses were performed using an Olympus FluoView 1000 inverted laser scanning confocal imaging system (Olympus, Japan). For EGFP detection, excitation was at 473 nm (diode laser) and detection between 515 and 535 nm. For mCherry and OFP detection, excitation was at 559 nm (diode laser) and detection between 565 and 625 nm. The images acquired from the confocal microscope were processed using ImageJ (http://rsbweb.nih.gov/ij/).

### Measurements of ion contents in yeast cells and rice tissues

For the measurement of Na^+^ content in yeast cells, each transgenic line was cultured in liquid synthetic complete (SC) medium at 30 °C. When the OD_600_ reached 0.6, 10 ml of the cultured solution was collected into a 15 ml centrifuge tube as a 0 h control sample. Then, each cell line was washed and cultured in liquid SC medium supplemented with 25 mM NaCl at 30 °C. At each time point, samples were collected as described above. All samples were washed with sterilized ultrapure water three times and dried at 65 °C for a few days. The samples were then digested using ultrapure nitric acid (Kanto Chemical, Japan) for 1 day. Digested samples were boiled at 95 °C for 10 min three times, and ion contents were determined using an inductively coupled plasma optical emission spectrometer (ICP-OES; SPS3100, SII Nano Technology Inc., Japan).

For the measurement of ion contents in rice tissues/organs, each sample was harvested and washed with ultrapure water twice. All samples were dried at 65 °C for 3–7 days, and the samples were digested with ultrapure nitric acid for a few days. ICP analyses were performed as described above.

### ^22^Na^+^-imaging using rice plants

Plants were hydroponically cultured using the plant growth chamber under 8 h/16 h light/dark cycle at 30 °C. The salt-stress treatment was started 2 or 3 days before the ear-emergence using the hydroponic solution containing 25 mM NaCl. Three days later plants were transferred onto the solution containing 50 mM NaCl and grown for another 5 days until flowering.

For ^22^Na^+^ absorption, the inflorescence was cut just above the node I and the cut-end was soaked in the solution containing 15 mM KCl, 1 mM MgSO_4_, 1 mM Ca(NO_3_)_2_, 0.5 mM NaH_2_PO_4_, and 1 kBq/ml ^22^NaCl for 30 min in the plant growth chamber. Then, the peduncle and the ear were separated at the bottom of the rachis base. The tissue distribution of ^22^Na^+^ with the resolution of 100 μm was recorded by a FLA-5000 image analyzer (*FUJIFILM* Co., Ltd.) with an imaging plate (BAS IP MS, GE Healthcare Lifescience). The line profile data of ^22^Na^+^ along the peduncle was obtained by setting the long narrow region of interest (ROI) on each sample. Then, the ROI was divided by the actual length of the peduncle (mm) or equally into 100 pieces irrespectively of the length and the ^22^Na^+^ signal value in each part was calculated.

### Availability of supporting data

All the supporting data are included as additional files in this manuscript.

## Additional files

Additional file 1: Figure S1. Expression profiles of *OsHKT1;4* in Nipponbare plants in the vegetative growth stage under salinity stress. (TIF 191 kb) Additional file 2: Figure S2. The production of *OsHKT1;4* RNAi lines and phenotypic analysis in the vegetative growth stage. (TIF 268 kb) Additional file 3: Figure S3. Ripening grains from *OsHKT1;4* RNAi plants accumulate more Na^+^ under salinity stress. (TIF 291 kb) Additional file 4: Figure S4.
^22^Na^+^-tracer analysis on peduncles of *OsHKT1;4* RNAi and Nipponbare WT plants, upon which salinity stress was imposed. (TIF 6988 kb)
