# Supplementary figures and images for: OsHKT1;4-mediated Na+ transport in stems contributes to Na+ exclusion from leaf blades of rice at the reproductive growth stage upon salt stress
Source: BMC Plant Biol. 2016 Jan 19;16:22. doi: 10.1186/s12870-016-0709-4 (PMC4719677; doi:10.1186/s12870-016-0709-4)

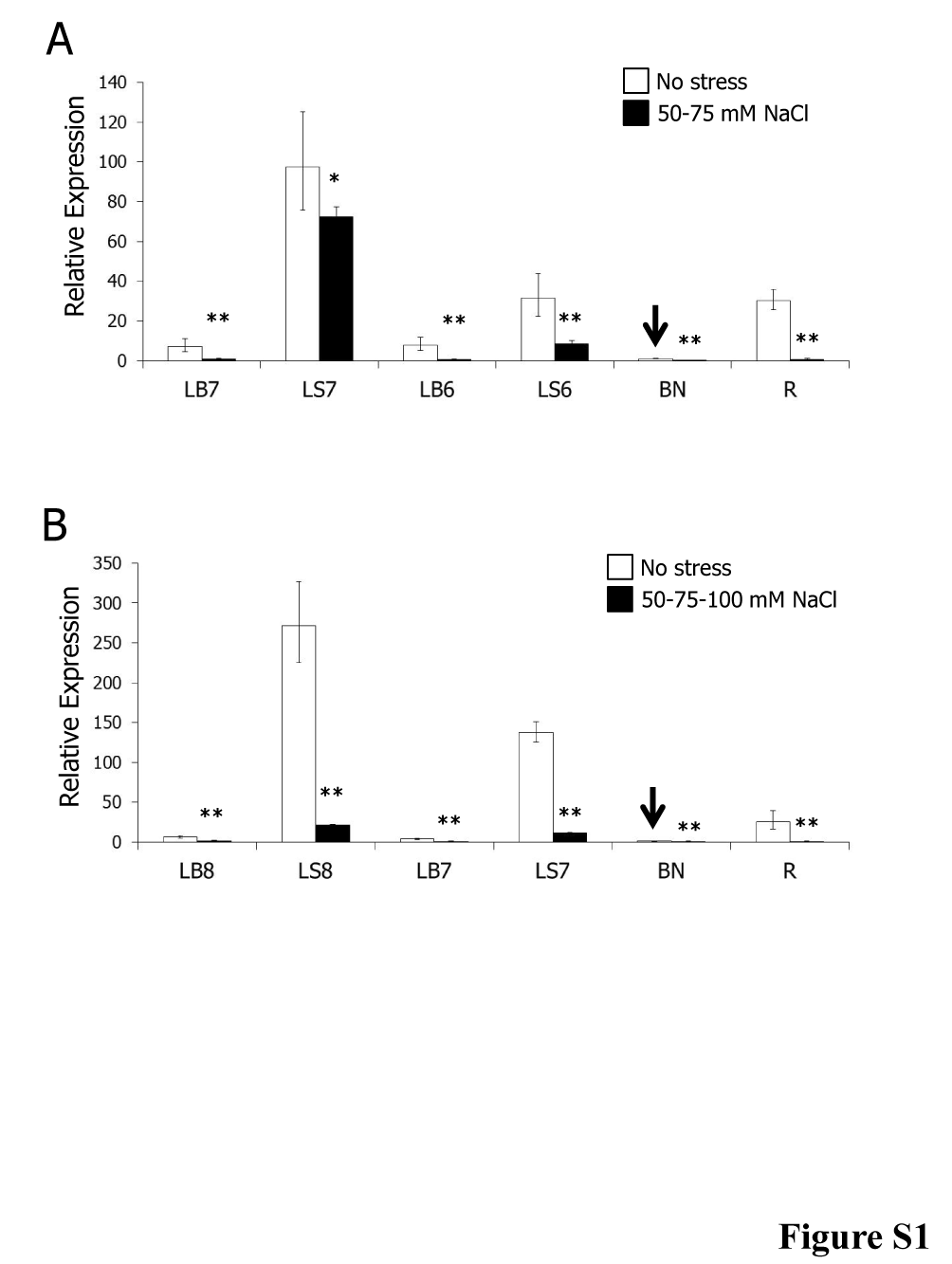

Supplement: Additional file 1: Figure S1. — Expression profiles of OsHKT1;4 in Nipponbare plants in the vegetative growth stage under salinity stress. (TIF 191 kb) [file 12870_2016_709_MOESM1_ESM.tif]

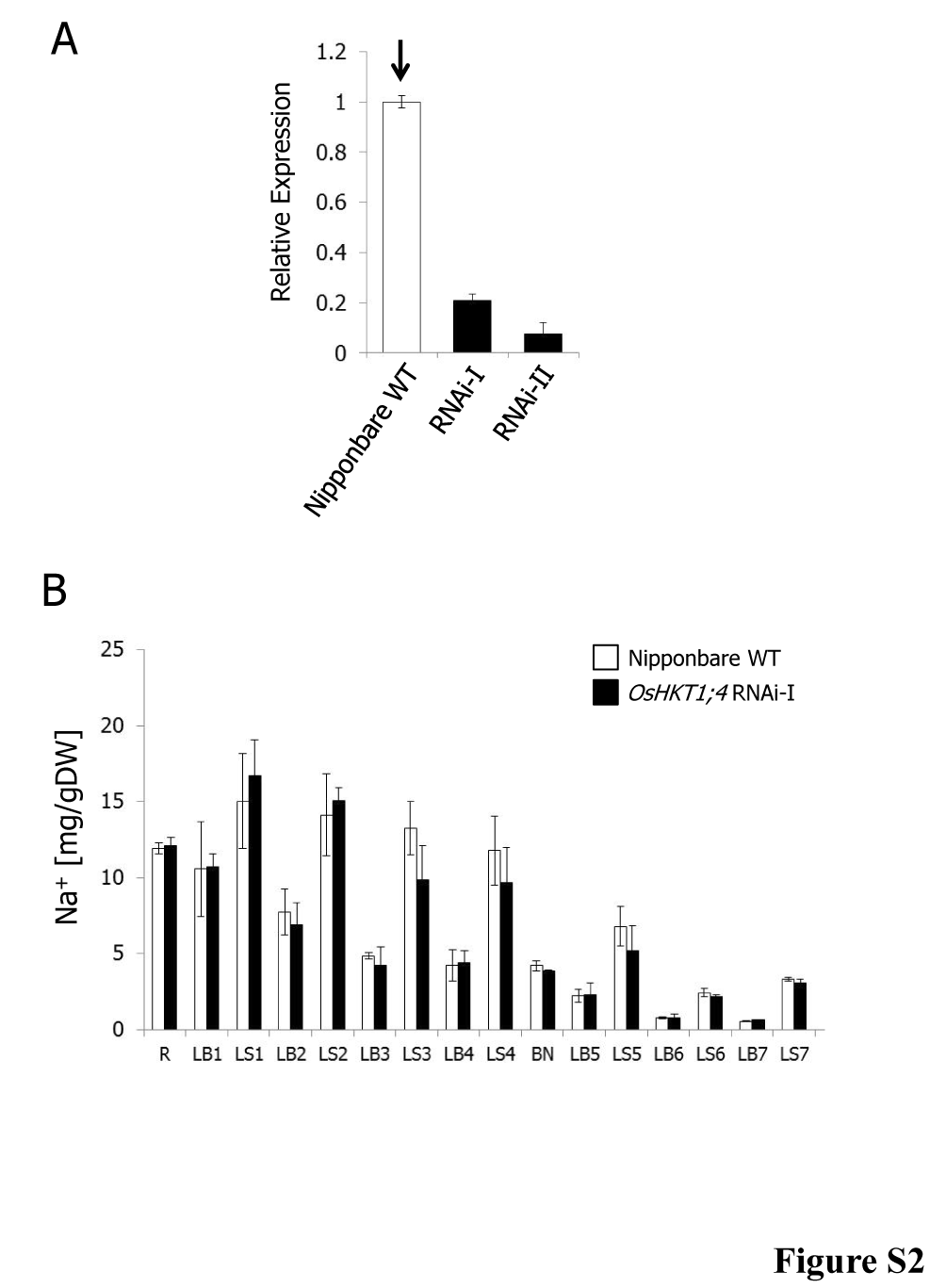

Supplement: Additional file 2: Figure S2. — The production of OsHKT1;4 RNAi lines and phenotypic analysis in the vegetative growth stage. (TIF 268 kb) [file 12870_2016_709_MOESM2_ESM.tif]

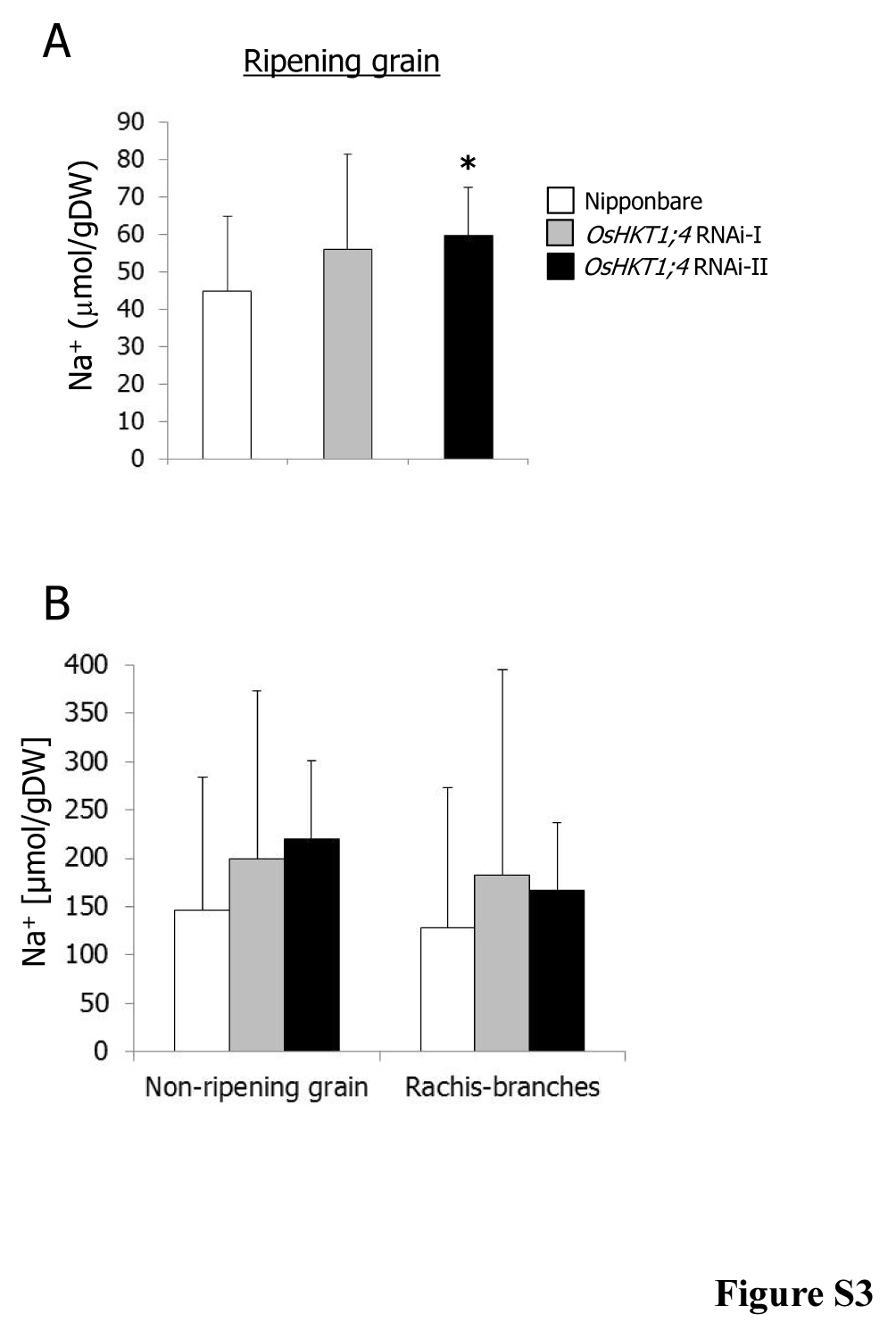

Supplement: Additional file 3: Figure S3. — Ripening grains from OsHKT1;4 RNAi plants accumulate more Na+ under salinity stress. (TIF 291 kb) [file 12870_2016_709_MOESM3_ESM.tif]

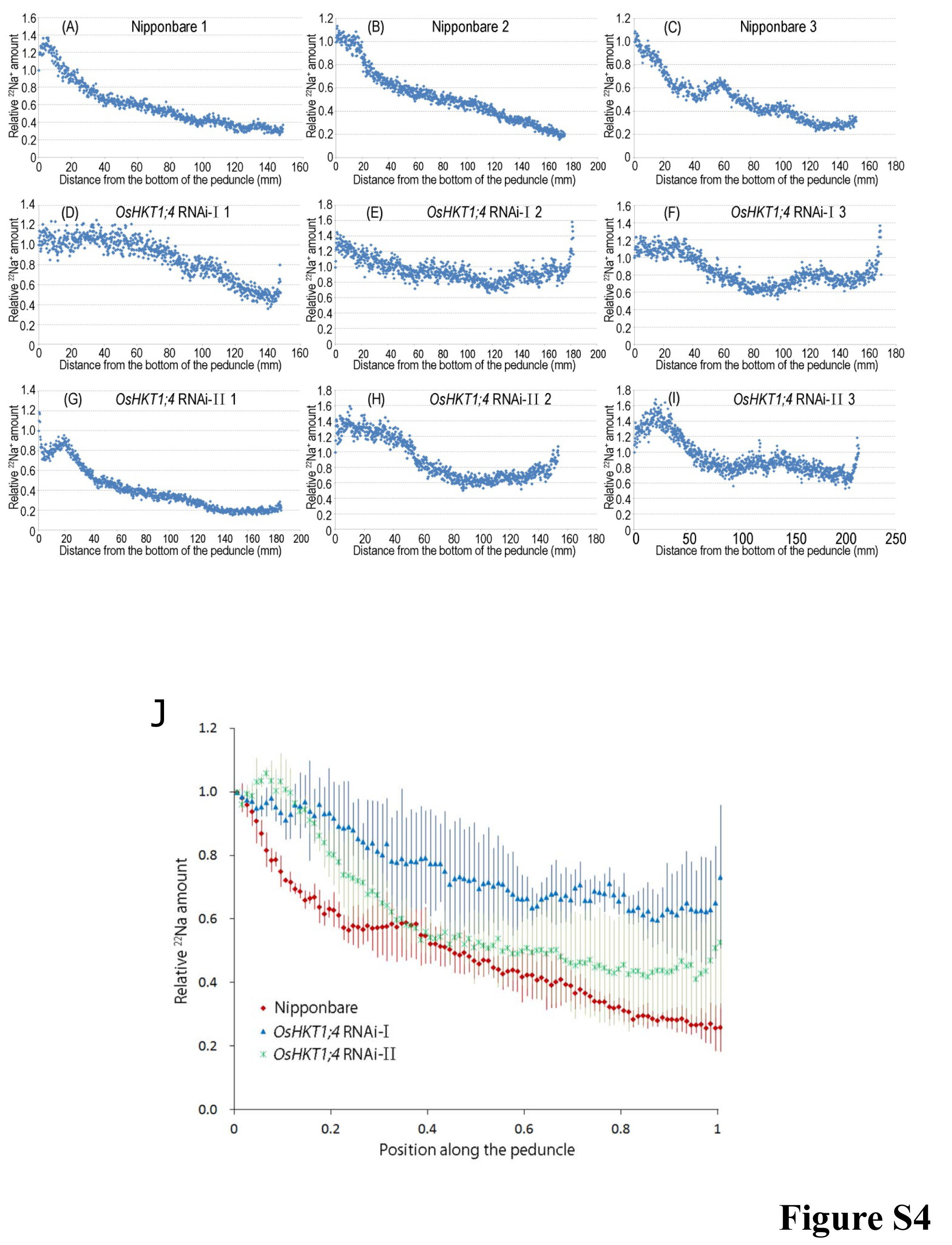

Supplement: Additional file 4: Figure S4. — 22Na+-tracer analysis on peduncles of OsHKT1;4 RNAi and Nipponbare WT plants, upon which salinity stress was imposed. (TIF 6988 kb) [file 12870_2016_709_MOESM4_ESM.tif]
